# The Intersection of Gender, Culture and Society for Caregivers of Older Adults Ageing in Place in Ontario, Canada

**DOI:** 10.1111/hex.70259

**Published:** 2025-04-14

**Authors:** Danielle Jacobson, Tashani Parker, Lauren Cadel, Elizabeth Mansfield, Kerry Kuluski

**Affiliations:** ^1^ Institute for Better Health Trillium Health Partners Mississauga Canada; ^2^ Dalla Lana School of Public Health University of Toronto Toronto Canada; ^3^ Leslie Dan Faculty of Pharmacy University of Toronto Toronto Canada; ^4^ Department of Occupational Science and Occupational Therapy University of Toronto Toronto Canada; ^5^ Institute of Health Policy, Management, and Evaluation University of Toronto Toronto Canada

**Keywords:** ageing at home, ageing in place, caregiving, gender norms, older adults, sexism

## Abstract

**Background:**

It is reported that women are more likely to be caregivers than men, experience a higher burden of care and increased emotional health sequelae as a result. Social location (a person's gender, culture, ethnicity, etc.) is known to influence caregiving experiences. However, there is limited work that draws attention to how cultural and linguistic diversity shapes the experiences and expectations of informal caregivers.

**Objective:**

The authors aimed to study how to reallocate health and social service resources to better support older adults ageing in place. However, some participants felt strongly about the role of gender. This report addresses the gap for better understanding (1) how gender influences informal caregiving for older adults ageing at home in Ontario, Canada, and (2) how culture may influence gendered caregiving expectations for this population.

**Design:**

A critical social justice paradigm and balance of care framework guided the research. Focus groups (15) and one‐one‐one interviews (7) were carried out. A collaborative approach to codebook thematic analysis was conducted.

**Setting and Participants:**

This study was carried out in Peel, a diverse region in Ontario, Canada. 42 individuals participated in the study (14 older adults, 10 caregivers and 18 healthcare providers).

**Findings:**

Four themes were found regarding the role of gender in caregiving: (1) women caregivers as catalysts for ageing in place, (2) gender norms, generational standards and the societal expectation for women to be caregivers, (3) the intersection of culture and gender on caregiving for older adults and (4) health service workforce as women‐dominant and linguistically diverse.

**Discussion and Conclusion:**

Service needs not currently met by Canada's healthcare system often become absorbed by women caregivers who facilitate ageing in place. Further research is required to better understand: (1) how a larger breadth of communities experience the intersection of gender and culture in the care of older adults in Ontario, Canada, and (2) how to better harness the diversity within Canada's homecare workforce to allow for cultural, linguistic and/or gender alignment with older adult clients.

**Patient or Public Contribution:**

Patients and caregivers were research participants; however, the focus groups were co‐design sessions, in which participants built and shaped personas and care packages.

## 1 Introduction

Statistics Canada [1] reports that approximately one‐fourth of Canadians engage in unpaid caregiving of older adults, with women being 42% more likely to be caregivers (also known as informal caregivers, carers, or unpaid caregivers) than men [2]. It is age‐old news that women are often societally expected to, and thus, engage in caregiving roles for older adults more often than men [3, 4]. As a result, women are reported to experience a higher burden of care and increased emotional health sequelae [5, 6, 7, 8]. While some research shows that men are increasingly engaging in caregiving work, women nonetheless still currently comprise the majority of caregivers [9] and engage in more caregiving hours [2]. Perhaps due to societal gender norms which position women as ‘feminine’ and ‘nurturing’ [10], care tasks also often differ by gender; while men have been reported to engage more frequently in maintenance tasks, women have been reported to perform personal care, scheduling, medical care, and emotional support [1].

It has been noted that caregiving experiences are heterogeneous and influenced by social determinants other than gender, such as culture [11, 12]. In the context of the current study, culture is conceptualised as the values, behaviours, and norms of a particular group of individuals. Despite the acknowledgement of the ‘importance of social location’ (a person's gender, culture, ethnicity, etc.) on caregiving experiences ([13], p. 13), there is limited evidence that draws attention to how cultural and linguistic diversity shapes experiences and expectations of informal caregiving [14], especially in the context of the care of older adults. Gender, race, ethnicity, and immigration are some examples of elements of social location that have been found to hold particular significance for caregivers' experiences caring for older adults [13, 15, 16, 17]. For example, one study conducted in Ontario, Canada, found that the intersection of gender and immigration for informal caregivers fostered difficulty in accessing services since they were ‘squeezed between the expectations of care from their home countries and the demands of caregiving in a Canadian context’ ([18], p. iii). Similarly, a Dutch study found immigration status to increase caregiving difficulty via a ‘triple burden’ involving culture, social status, and practical barriers [19].

The current study, therefore, addresses the gap for a better understanding of (1) how gender influences informal caregiving for older adults ageing at home in Ontario, Canada, and (2) how aspects of culture and immigration may influence gendered caregiving expectations for this population.

## 2 Methods

### 2.1 Aim

The authors entered into the research aiming to study how to reallocate health and social service resources to better support older adults (65 years of age and older) ageing in place. Participants were asked about the type of support that members living in diverse communities may have and would need access to in order to age in place. In discussing caregiving supports, some participants felt strongly about the role of gender and communicated in‐depth, rich thoughts. This report, therefore, centres on the role of gender in caregiving in the context of ageing in place. More details on the methods of this study may be found in the larger report [20].

### 2.2 Conceptual Framework and Positionality

A social justice paradigm guided the research, influencing design, data collection, analysis, and knowledge mobilisation [21]. Epistemologically, the authors view knowledge as value‐mediated, and ontologically, social structures are viewed as having an impact on individual experiences [22]. The balance of care framework also guided the research. This framework, in particular, guides researchers to establish the best possible mix of resources to meet the needs of the population in a specific area’ ([23], p. 81). The balance of care framework facilitates comprehension about how to reallocate health and social resources and has largely been applied in previous research to ageing populations [23, 24, 25, 26, 27, 28, 29]. Throughout the research process, the research team discussed the ways in which their positionality had an impact on the research [30, 31]. For example, the team discussed experiences of privilege and marginalisation, motivation to improve experiences of ageing at home for older adults, and academic background and training, which influenced how each member interpreted the data.

### 2.3 Study Setting and Participants

This study took place in Peel Region, Ontario, Canada. This region is diverse [32], with nearly half of the population being immigrants [33] and older adults being the most rapidly rising group [34]. 42 individuals participated in the study (14 older adults, 10 caregivers, and 18 healthcare providers). Participants completed a socio‐demographic questionnaire (see Table 1: Participant socio‐demographics). When asked about sex *and* gender, some older adults became uncomfortable, defensive and/or confused (e.g., thought the same question was being asked twice). In such instances, the researchers explained the difference between sex and gender (sex referring to biological characteristics and gender referring to social identity) and the importance of collecting this information. The authors will continue to rethink how progressive demographic questions may be most appropriately addressed with older adult participants given that, often, the conceptualisation of sex and gender with which they grew up differs from current norms.

**Table 1:** Participant socio‐demographics (patients, caregivers and healthcare providers [20]).

Age	Sex	Gender identity	Racial or ethnic identity
30–39: 6	Male: 4	Man: 5	Asian mixed heritage: 1
40–49: 8	Female: 38	Woman: 37	South Asian: 4
50–59: 7			Central Asian: 1
60–69: 9			East Asian: 1
70–79: 11			Chinese: 2
80–89: 1			Middle Eastern: 5
			‘Mix’: 2
			Bi‐racial: 1
			White: 9
			Latin American: 1
			South American/West Indian: 1
			West Indian: 1
			Black: 9
			Caribbean: 2
			Jamaican: 1
			Southern European: 1

### 2.4 Recruitment and Consent

This study was approved by Trillium Health Partners research ethics board (ID#1095). Purposive recruitment from September 2023 to February 2024 centred around building relationships with organisations that serve diverse communities in the Peel region. The research team attended meetings of diverse community organisations to share study details and listen to the community's experiences and perspectives on the approach. Some participants were recruited to engage in the study from these meetings. Participants had to either be an older adult or have cared for an older adult (informally or formally) and be cognitively able (i.e., have the capacity to provide informed consent). All participants identified as having the cognitive ability to consent, and researchers did not identify otherwise. However, if an individual was unable to consent, they would have been included in the study with the support of a substitute decision‐maker. English speaking was not a requirement, and interpreters were offered when participants preferred to participate in their mother tongue. Community organisations, collaborators and the researchers' networks helped to distribute study information. Potential participants contacted members of the research team to indicate their interest and learn more. Participants were provided with an informed consent form and consented to participate either by signing the form or verbally (audio recorded).

### 2.5 Focus Groups and One‐on‐One Interviews

This study was part of a broader sequential mixed‐methods research programme. Using quantitative methods (reported separately), administrative health data (Resident Assessment Instrument—Home Care data) from Mississauga residents (a city within Peel Region) on long‐term care waitlists was segmented by five MAPLe levels. MAPLe level, otherwise known as a method for assigning priority levels, is a designation which indicates an individual's ability and ranges from level 1 to level 5 [35]. MAPLe level 1 would designate an individual with a high degree of independence, whereas MAPLe level 5 would indicate an individual with a high degree of need for support [35]. Data garnered per MAPLe level was then written into five characterisations called risk profiles, which were presented to participants during focus groups and interviews and then built upon and refined via co‐design into personas (portrayals or hypothetical representations illustrating an individual and what they might experience; see Table 2: Persona example) [36, 37, 38]. Participants co‐designed the personas by adding information not captured about what that person may experience and by indicating where descriptions of the persona needed to be tweaked or even eliminated. After building on the personas, participants co‐designed a care package based on what that persona would need to age in place (see [20] for a more in‐depth account of the creation and use of the personas and care packages). 15 focus groups and seven one‐on‐one, semi‐structured interviews were conducted via Zoom communication software [39] and were audio and video recorded. Older adults and caregivers participated together (without healthcare providers), and healthcare providers participated together (without older adults and caregivers). Participants were invited to engage in multiple sessions to allow enough time to share in‐depth experiences and to encourage co‐design, iterative learning, and comfortability [40]. Six individuals participated in a second focus group, three in a third focus group, and two in a fourth. One focus group was facilitated by an interpreter. Each participant was compensated with a $50 CAD gift card.

**Table 2:** Persona example.

MAPLe Level	Persona description
**4**	Khalid is an 88‐year‐old man and speaks Arabic as his first language. He lives in a house with his wife (Layla) in a low‐income neighbourhood and struggles to meet his daily living needs. Layla provides support; however, she is older and experiencing frailty and stress in her caregiving role. She is ignoring her own ailments and also needs support. Khalid and Layla find it difficult to manage on their own, and neighbours have been unable to help. Layla has noticed that Khalid is showing signs of short‐term memory problems, which impacts his ability to make decisions about daily activities and make himself understood. However, Khalid can recognise different times of day in his usual environment and people who are familiar to him. He uses the telephone independently but needs to wear hearing aids, which he sometimes forgets to use. He independently uses the toilet, though with difficulty due to limited mobility in his joints. He needs cueing for personal hygiene and help with bathing (e.g., washing his lower body, but actively participates), dressing and managing medications (requires blister packs). He can independently feed himself most of the time, though sometimes he has difficulty. Khalid relies on others to prepare and heat meals, grocery shop, complete housework (including laundry) and manage finances. Khalid walks with difficulty, sometimes struggling to get in and out of bed and chair, and has had a fall in the last month. Khalid now requires assessment for a gait aid. He now uses non‐emergency ambulances as he needs to be transported on a stretcher for appointments and is able to see his family doctor, who does home visits when needed. Khalid prefers services provided in Arabic and providers familiar with his cultural practices (e.g., times of prayer). Sometimes he feels lonely as he struggles to go out on his own to visit family and friends and also struggles to afford day programmes. Khalid needs someone to help him engage in faith‐based activities in person and online. Khalid and Layla's children provide emotional support but do not live with them, making it difficult to care for them in a timely manner.

### 2.6 Data Analysis

Focus group and interview recordings were transcribed verbatim, with identifying information removed. Transcripts were checked against the audio recording to ensure accuracy. Codebook thematic analysis [41, 42, 43] was conducted using NVivo qualitative data analysis software [44]. D.J., J.B., and T.P. each independently coded three transcripts before coming together to review codes. With a majority of overlapping meaning and scant disagreement, D.J. independently coded the remaining transcripts. Disagreement was reconciled through analytic discussions. J.B. and T.P. each reviewed the codes on half of the remaining transcripts. Few codes were added and none were decoded; additions were made both to existing codes to add nuance and also to ensure clarity across codes. D.J. proceeded to conglomerate codes with overlapping meanings and collapse codes no longer relevant to the developing analysis. D.J. and T.P. discussed emerging themes, which were refined in relation to the original research question. Overall analysis and study findings were presented to and validated by community members including study participants and also non‐participants part of partner community organisations. Selection and formation of themes included in this manuscript were guided by participants' emphasis on the importance of gender within the caregiving role; each participant type (i.e., older adults, caregivers, and healthcare providers) communicated in‐depth, rich thoughts regarding this facet of the experience.

## 3 Findings

Participants (older adults, caregivers, and healthcare providers) in this study, which took place in a diverse region in Ontario, felt that gender played an important role in the caregiving experience for older adults. Five themes surrounding the intersection of gender, culture, and society for informal caregivers of older adults ageing in place were found and are described below: (1) women caregivers as catalysts for ageing in place, (2) gender norms, generational standards, and the societal expectation for women to be caregivers, (3) the intersection of culture and gender on caregiving for older adults, and (4) health service workforce as women‐dominant and linguistically diverse.

**Theme #1: ‘If you could rent a daughter, that would avoid the need for most long‐term care patients’: Women caregivers as catalysts for ageing in place**

Participants described that women caregivers were important catalysts to ageing in place. Gabriel (geriatrician) described that ‘what Jean [persona] really needs … is someone to care about them’, however, ‘that higher level of help … is not accessible anywhere in our [healthcare] system’. He explained that often it was a woman informally providing that ‘higher level of help’ to support ageing in place:

I have a mentor who would always say, ‘What are the two keys to successful ageing? Become exceedingly wealthy and have an unmarried daughter’. … That's kind of like sexist, I think, but you just need someone who's going to be with you and care for you.

Clara (social worker) similarly explained that ‘it's usually the daughters and daughter in laws or some female relative of sort, nieces, whoever, that tend to be the hands on caregivers’. Women in caregiving roles were so ubiquitous that many participants spoke with humour about the concept of ‘rent‐a‐daughter’, which indicated that if an older adult had a wife or daughter, they were able to age in place longer and more effectively. James (geriatrician) explained that ‘they should have a company called “rent‐a‐daughter” because if you could rent a daughter, that would avoid the need for most long‐term care patients’. She said that ‘if you have a daughter, if you have a wife, you can stay at home forever’; however, she questioned, ‘but is that fair to the wife or daughter?’ Akeelah (recreation therapist) used the same language to denote this concept:

In our [specific] program the [healthcare provider] often says ‘I wish there was a resource, a company, an organization where you could like rent‐a‐daughter for a day or like for a month’, where when family can't be there you can find someone to go in and do that work with the patient in their home.

Gabriel (geriatrician) clarified: ‘Why is it always a daughter? Again I think that's just a stereotypical, like women are more carers than men are. And if you look historically, if you look at my patient roster, the primary contact is often the daughter. I mean almost always, really’.

Some participants explained that often women caregivers prioritise the care of others before their own care. Mai (older adult) explained: ‘She [the persona] has to look after her husband and her kids and then she can look after her[self]’. Francesca (caregiver), Sunita (caregiver) and Trinh (caregiver) similarly engaged in a discussion during one focus group about the ubiquity of women assuming caregiving roles, being catalysts for ageing in place:

Francesca: Why is it, why again, as women, and we're all women here today, we are everybody else's primary caregiver? Who is caring for the caregiver?

Sunita: No one.

Francesca: … And we assume that role and all the work that goes with it. … My husband's parents were failing and who looked after them?

Sunita: You did.

Trinh: Yeah, it's women.

**Theme #2: ‘It's an equity issue of the expectations that are on women caregivers’: Gender norms, generational standards, and the societal expectation for women to be caregivers**

Gender norms and societal expectations for women to be caregivers were described, particularly surrounding general expectations from families and providers, expectations from older adults about who would do the caregiving, and the impact of these expectations on caregivers' comfort level. Participants like James (geriatrician) discussed ‘the expectations that are on women caregivers’ as ‘an equity issue’, which she thought ‘brings up … large system issues but also sexism issues’. Francesca (caregiver) conveyed the ubiquity of women being expected to carry the entire burden of labour and men being unexpected to contribute to caregiving work:

The other thing too that I encounter, and this is at my doctor's office, someone will say, … ‘how come you're doing it all [caregiving work] yourself?’ And to be very blunt, … the simple word of saying, ‘Oh, I have brothers’ is enough for everybody to go, ‘Oh! Okay.’

She said that there was a gendered ‘expectation by the medical staff that we are [the caregivers] as the women’ and that her ‘brothers were useless. And if not useless, difficult … adding additional [caregiving] roadblocks’.

Camila (older adult/caregiver/nurse) also described the pressure of being expected to be the caregiver as a woman despite having male siblings: ‘[Parent] had a problem with her [condition]. So, whenever that happens, she calls me and I run. Meanwhile, my brother lives 10 minutes away, he never goes’. Gabriel (geriatrician) echoed that ‘the sons do not help at all. They have no interest in it. I've had families where the daughter is a high‐level executive, and the sons are retired. And still all the care is falling to the daughter’. Akeelah (recreation therapist) elucidated that one contributing factor to the expectation of women as caregivers was the role of generational gender norms and older adult's expectations of who does the caregiving: ‘Given the generation of who our geriatric patients are, when they were younger, what was their gender role expectation? … How do they interpret what's appropriate? … If it comes to personal care … maybe more comfort when it's a female’. She continued to explain how gendered caregiving expectations manifested in her own life: ‘My dad was like, “… if I ever get sick you're just going to move home and take care of me”. I'm like, “okay”. I have two older brothers [laughs]’. James (geriatrician) explained that traditional gender norms also influenced the self‐care tasks that some older men were and were not accustomed to completing:

Sometimes … someone comes into hospital and has just completely fallen off the rails. And then when you get the story, it's because their wife passed away. … Their wife was taking care of all of these things. … For some of the older patients … they've never had to make their meals, they've never had to do any of this stuff, never had to do laundry.

Gender norms also had an impact on caregivers' comfort level. Priya (palliative care physician) said that there may be a ‘discomfort with certain elements of care, personal care. … Daughters tend to be more comfortable with those activities than sons’. However, Maria (palliative care physician) said she had ‘seen many sons do a great job’. Akeelah (recreation therapist), too, had ‘seen some extraordinary sons come through’, but saw ‘more daughters being hands‐on than the sons’, which was ‘just traditional gender roles in society’.

Caregiving was discussed by participants like James (geriatrician) as a skill set at which women were perceived to more naturally excel, ‘tied to capability’:

I do see women, whether they've been socialised or whether we do have a natural inclination. … But they intuitively understand much more about how to care for someone as needs are increasing. And they also seem to recognise it a lot better too. … Women, the daughters, or the wives [are] seemingly more capable.

Clara (social worker) explained that often, men lacked that ‘caregiving kind of skill set’ and ‘intuition’ and thus experienced higher levels of caregiving stress, requiring more ‘health teaching and education’:

The son's caregiver stress probably would be much higher, to be very honest, because the intuition of caregiving isn't necessarily there. … And level of frustration and just need for more health teaching and education. A lot of it is prompting the male caregiver … they don't know what they don't know. So sometimes giving different scenarios is helpful to help him see like, ‘oh, yeah, that does happen’. Because he may not know to ask. … He may not intuitively again remember to bring that up [with the provider].

Clara further compared gendered behaviours, which contributed to men's perceived lacking caregiving skill set:

Women are used to asking for help more. Or at least voicing the concerns and issues more. Where men are like, they focus on the objective stuff. And they don't necessarily see the whole picture in the same way or won't convey it all initially. So we have to do a little bit more digging and probing.

James (geriatrician) too noticed ‘high level patterns’ that men often ‘struggled when they're trying to get more help’. Another purported reason proposed by Clara for this perceived lacking skill set was perhaps because many men ‘may not have been a caregiver to children or anything to see how that variability happens’.

**Theme #3: ‘With our different immigrant populations … usually, you'd have more individuals and more females involved’: The intersection of culture and gender in caregiving for older adults**

Discussion of societal expectations of women versus men's caregiving role extended to cultural considerations. Participants had divergent views about how culture affected the gendered role in caregiving work. Binh (HSW; home support worker) explained that in some cases, the reduced expectation for men to engage in caregiving work was rooted in culture:

In my family, it's me. I'm going to be the primary caregiver. My brother shows up twice a year, and he's the prodigal son. That happens in a lot of different cultures. … Someone else there that could step in and help … would be one of the easiest ways at least to alleviate some of the stress.

James (geriatrician) agreed that ‘in people from other cultures … [for] women, the daughters, or the wives … the expectation [of caregiving] seems to be very much embedded in their life’. However, other participants, like Akeelah (recreation therapist), described that in other cultures, sons were pivotal in providing wraparound care:

With our South Asian, like our Indian patients that come through, their families really wrap themselves around the patient, whether it's son or daughter. … So I would say that stands out for me and maybe it's just because of the number of those patients I see, but those sons really take care of their family.

Clara (social worker) explained that the increased wraparound care in certain cultures was more feasible with larger family sizes, which were perceived as more common in immigrant populations, but that the care did tend to fall onto the women: ‘Especially with our different immigrant populations, there would be more of a community support. Many larger families. … Usually, you'd have more individuals and more females involved’. Aisha (OT; occupational therapist) detailed that sons may be more involved caregivers in some cultures since a woman engaging in personal care for her father may be considered ‘taboo’: ‘I can't imagine she's [daughter] doing his [persona's] personal care because culturally that role of father‐daughter is almost a little taboo to help with that personal care to that degree’.

Ayaan (OT) explained that in particular cultures, it was commonplace for older adults to prefer formal and informal caregivers of the same gender:

It does happen though. … I think in the Muslim community that happens, where a male

would only want a male and wouldn't want a female. But in general, you hear it more from what I've heard with my own ears, is that we do have instances where … female patients are asking for female nurses. Or even PSWs. … And if there's a male they would want someone else with them.

Lin (caregiver), Jan (caregiver), Esha (caregiver) and Benilda (caregiver) detailed in a discussion during one focus group that part of the preference for same‐gender caregivers had to do with values related to modesty:

Lin: As a son, it's kind of difficult for him to maybe care for her [his mother] in certain ways being that it's his mom and there's, you know, a sense of modesty, embarrassment. If she has an accident [incontinence] and he has to like clean her or something like that.

Esha: … That is so true. …

Benilda: That's right.

Jan: Yes. My mom went through that because she would not allow my son—my brother was helping to take care of her and she didn't want him to bathe her. Because for her it wasn't proper.

Esha: That's right.

**Theme #4: ‘It tends to be minority women in these roles’: Health service workforce as women‐dominant and linguistically diverse**

While participants mostly focused discussion on the role of gender for informal caregivers, some participants pointed to the reality that often women and, in particular, minority women engaged in service provider roles for older adults ageing in place. Navdeep (OTA/PTA; occupational therapy assistant/physical therapy assistant) explained that ‘nursing, you can see that almost every unit is a lot more female based than it is male based’. He clarified that ‘it's not to say anybody is more qualified or anybody does it better or anything like that. It just … is definitely more female dominated’. Ayaan (OT) said that ‘OT as well is more female dominant. … In school, it was in a 60‐student class there's only five males’. Akeelah (recreation therapist) explained that the women‐dominant health service workforce was a manifestation of ‘our traditional conditioning of healthcare’.

Priya (palliative care physician) highlighted that it was often racialised women in particular in PSW roles and that English was not always the ‘helpful intermediate’ to communicate with older adults, demonstrating the importance of linguistic alignment or tools to support communication:

It's more often the native English speakers that complain … expecting a native English speaker. … But I think it adds that extra layer that if … she's [persona] speaking, I think it was Cantonese, and if her provider primarily speaks entirely different language, again that's where I think pictorials can help. It's not always English that's going to be the helpful intermediate. And recognizing that a lot of our PSW workforce are racialized providers who may be newer to Canada and are also figuring that out. … It tends to be minority women in these roles.

Jayden (nurse) explained that it was near impossible to procure a service provider with the alignment of ‘gender, and language, and culture’:

We can't get culturally equal people going to homes. It's the craziest thing. Because makes common sense to me that you would send somebody of speaking language to the home and make that a priority. … Sometimes we need males and then they send females.

Navdeep (OTA/PTA) clarified that these requests for cultural, linguistic and/or gender‐aligned healthcare providers ‘makes it difficult on some of the companies’ since ‘you only have so many people in the roster to kind of pull from’ and that often services ‘can't follow through with that request’; ultimately, ‘it's hard to find somebody from another gender to kind of go and do the same role if somebody makes a request for it’.

## 4 Discussion

Participants in the current study, who spanned multiple generations, perceived women caregivers as catalysts for ageing in place. The ubiquity and importance of women's caregiving role was expressed through the concept of ‘rent‐a‐daughter’—an imagined service to support older adults ageing in place when a woman relative was unavailable. While the authors are not aware of scholarly work that references the ‘rent‐a‐daughter’ concept, some private home‐care services in the United States market themselves with this idiom. ‘Rent a Daughter’ service in Beachwood, Ohio, offers all‐hours support of activities of daily living (e.g., incontinence), instrumental activities of daily living (e.g., laundry) and beyond (e.g., pet care) [45]. Another company by the same name with similar services exists in Wisconsin [46]. This higher level of service (e.g., assistance with instrumental activities of daily living) is not currently covered by the Ontario Health Insurance Plan and is only available privately, making it inaccessible to most Ontarians, despite existing need [27, 47, 48]. Since the majority of informal care is often provided by women, service needs not currently provided by the healthcare system become absorbed by this group, who facilitate ageing in place on an unpaid and often unrecognised basis [49].

Aligned with previous research, this study highlights the societal expectations foisted on women to engage in such caregiving roles [3, 4, 6, 7, 10, 50]. While some research exists on adult caregivers' gender role attitudes and impact on the care provided [51], to the author's knowledge, novel to the current study is the finding that generational gender norms influenced older adults' expectations about who engaged in caregiving work and tasks to which they themselves were or were not accustomed to carrying out (e.g., cooking, laundry, etc.). For example, older adult men were perceived to have grown up with the expectation that women did the caregiving and household work and therefore lacked these skills, posing difficulty upon the death of a woman spouse. González et al. [52] highlight that women's higher caregiving burden may be elucidated by this ‘persistence of traditional gender norms in addition to prevailing gender relations in the labour market’ and recommend a policy approach to address gendered caregiving norms and to work toward equitable distribution of caregiving work (p. 91).

The burden of care in which women were expected to engage was conveyed by participants as an equity issue, especially when held up against the lack of societal caregiving expectations and the burden of labour for men. Participants in the current study expanded on the intersection of gender and culture, describing that in some cultures, men were not expected to engage in caregiving work, while in others, they played a pivotal role in wraparound care to support older adults. In some instances, it was taboo for a woman to care for her father and like‐genders were preferred for personal care (e.g., at times, older adult men were perceived as preferring son caregivers and older adult women were perceived as preferring daughter caregivers). Similarly, one American study found that while women predominantly engaged in caregiving work, sons tended to provide more care for fathers and daughters more care for mothers, respectively [4]. Regardless of culture, women were consistently perceived as playing a dominant caregiving role.

In other studies, culture has also been found as a moderator for gender differences in informal care for older adults [10, 53]. For example, while women were conceived of as caregivers across cultures, Pharr et al. [54] highlight nuanced differences in different communities: in Asian American culture, gender and birth order intersected such that the youngest daughters specifically were expected to do the caregiving work; in Latin‐American culture, the oldest daughters were expected to engage in this role; in African American culture, caregiving work was often completed by both immediate family and extended family and friends, though still prescribed to women; and in European‐American culture, caregiving responsibility fell to women, though instead of a cultural attribution, this expectation was ascribed to personal responsibility. In the current research, some specific cultural communities' gendered conceptualisation of caregiving were discussed (e.g., South Asian communities' involvement of sons in wraparound care for older adults in addition to women's involvement); however, further research is required to better understand how a larger breadth of particular communities (across a range of generations) experience this intersection in Ontario, Canada, with implications for strengths‐based approaches to community care.

In the current study, it was found that women played a dominant caregiving role even when they had brothers more conveniently located to the older parent. Similarly, one American study found that women provided more care even when they had brothers [4]. It is possible that this unequal division of labour was rooted in some participants' perception of women as having a natural intuition for caregiving, also cited in the literature as women's ‘natural’ role as a caregiver [55]. Further, the unequal division of labour may relate to some participants' perceptions of men as lacking the natural caregiving skill set and, therefore, experiencing a higher degree of caregiver stress in addition to requiring more education and support to engage in the caregiving role. Parallel to this finding, Zygouri et al.'s [10] review on gendered caregiving experiences for older adults highlights studies that demonstrate that men often experience increased levels of stress compared to women regarding caregiving work given the shifting distribution of household labour and engagement in what was considered a feminine task [56]. However, other literature has reported higher stress levels in older adult women caregivers, specifically when caregiving for a spouse [57].

Minority, linguistically diverse women were perceived by participants in the current study to be dominant within the health service workforce, particularly PSWs; participants described that many older adults preferred, but did not receive, cultural, linguistic, and/or gender alignment with providers. Similarly, Zagrodney and Saks [58] highlight that most PSWs are minority women and that the occupation often provides low compensation and a lack of job security. It has been purported that this poor compensation and working conditions root back to formal, paid caregiving being ‘viewed as [an] extension of unpaid care work’ ([52], p. 91). Similar to participants in the current study, 62% of PSWs in an Ontario survey indicated that it was important to communicate with care recipients in their own language, indicating a preference for linguistic alignment with clients [59]. Despite PSWs being found to ‘reflect the ethnic and racial diversity of Ontario’ ([59], p. 4), it was often not possible for participants to be assigned culturally, linguistically, or gender‐aligned providers—this is an important limitation of Canada's current healthcare system. Further research is required to better understand how to harness the strength of diversity within Canada's homecare workforce to provide culturally and linguistically aligned care where possible.

## 5 Conclusion

While the current study took care to include a diverse sample of participants, not all populations in the study region were represented. Further research would therefore be beneficial utilising the same balance of care approach to better understand additional underrepresented communities not captured in the current study. Of benefit to this endeavour will be to strive to include diverse personnel on the research team (particularly who speak the same language as the communities involved in the research).

Given that informal care is often provided by women, service needs not currently met by Canada's healthcare system often become absorbed by informal women caregivers, who facilitate ageing in place. This study highlights how generational gender norms influence older adults' caregiving expectations and the related inequity in societal caregiving expectations and the burden of labour for women. While men of particular cultures were at times described as engaged in caregiving tasks, women were consistently perceived as playing a dominant caregiving role. Further research is required to better understand how a larger breadth of particular communities experience the intersection of gender and culture in the care of older adults in Ontario, Canada. A key limitation of Canada's healthcare system observed across the sample was the inability to accommodate cultural, linguistic, and gender alignment between providers and clients, despite having a workforce that reflected the Ontario population's diversity. A service priority was therefore demonstrated to better harness the asset of diversity within Canada's homecare workforce to strengthen home and community care and centre diverse older adults in their care.

## Author Contributions

**Danielle Jacobson:** conceptualisation, formal analysis, writing – original draft, investigation, writing – review and editing, project administration, validation, resources, visualisation. **Tashani Parker:** formal analysis, writing – review and editing, investigation, project administration, validation, resources, visualisation. **Lauren Cadel:** writing – review and editing, investigation, project administration, resources. **Elizabeth Mansfield:** writing – review and editing, funding acquisition. **Kerry Kuluski:** supervision, investigation, methodology, writing – review and editing, formal analysis, funding acquisition, conceptualisation, resources, visualisation, project administration.

## Ethics Statement

This study was approved by Trillium Health Partners research ethics board (ID#1095). All participants consented (either in writing or verbally with audio‐recording) before engaging in the study.

## Consent

All participants consented (either in writing or verbally with audio‐recording) prior to before engaging in the study.

## Conflicts of Interest

The authors declare no conflicts of interest.

## Data Availability

The data that support the findings are reported directly in this manuscript. The raw data (i.e., complete transcripts) are not publicly available due to privacy or ethical restrictions.
